# Case Report: Mast cell anergy: absence of symptoms after accidental re-exposure to amoxicillin/clavulanic acid 3 days after anaphylaxis

**DOI:** 10.3389/falgy.2024.1366922

**Published:** 2024-03-11

**Authors:** Loris Guyénard, Marie Tauber, Sophie Debord-Peguet, Frédéric Berard, Audrey Nosbaum, Florence Hacard, Mariana Castells, Jean-François Nicolas

**Affiliations:** ^1^ Allergologie et Immunologie Clinique, Centre Hospitalier Lyon-Sud, Hospices Civils de Lyon, Lyon, France; ^2^ Team Epidermal Immunity and Allergy, Centre International de Recherche en Infectiologie (CIRI)—Université Claude Bernard Lyon 1—Inserm U1111—CNRS—ENS, Lyon, France; ^3^ Service d’Anesthésie-Réanimation Civilo-Militaire, Hôpital Edouard Herriot, Hospices Civils de Lyon, Lyon, France; ^4^ Drug Hypersensitivity and Desensitization Center, Mastocytosis Center, Brigham and Women’s Hospital and Harvard Medical School, Boston, MA, United States

**Keywords:** beta-lactams, drug allergy, case report, skin test, systemic reaction

## Abstract

Empty mast cell syndrome, also named post anaphylaxis mast cell anergy (PAMA), is a temporary state of loss of mast cell responsiveness after a severe immediate hypersensitivity reaction. In this study, we describe a case of PAMA after accidental re-exposure to amoxicillin in a patient who developed severe anaphylaxis to this drug three days earlier in the operating room. To our knowledge, this report is the second to document this phenomenon.

## 1 Introduction

Postanaphylactic mast cell anergy (PAMA) defines a state of mast cell unresponsiveness following massive activation. It is manifested by a loss of cutaneous reactivity to skin tests and has been described in several series (1, 2) and clinical cases (3–6) in the context of anaphylaxis to hymenoptera venoms and perioperative anaphylaxis. Current recommendations by allergology societies indicate diagnostic skin testing between 4 and 8 weeks (7, 8) after a severe hypersensitivity reaction to avoid false-negative results. The mechanisms involved in this phenomenon are incompletely understood and implicate the depletion of mast cell mediators.

In this study, we report a case of mast cell anergy that enabled a patient to tolerate accidental reintroduction of amoxicillin, despite having presented with intraoperative anaphylactic shock to amoxicillin 3 days earlier.

## 2 Case report

A 77-year-old patient was admitted to the hospital for undergoing a left ureteroileoplasty. During anesthetic induction, he presented with hypotension after mechanical ventilation treated with epinephrine, from which he recovered, and when receiving prophylactic antibiotic therapy with 2 g of amoxicillin/clavulanic acid intravenously, he developed diffuse urticaria with bronchospasm and suffered a cardiac arrest within 2 min. A successful cardiopulmonary resuscitation was done, and the patient required 6 mg of intravenous epinephrine, external electric shock, 11 puffs of salbutamol, and 100 mg of hydrocortisone. Serum biomarkers were obtained and elevated tryptase and histamine levels confirmed mast cells and possibly basophils activation and degranulation during anaphylaxis (Table 1). The surgical procedure was not performed, and the patient was transferred to the intensive care unit for recovery and monitoring. Allergy to amoxicillin or clavulanic acid was suspected. On day 3 after the event, he developed pneumonia and 1 g of oral amoxicillin/clavulanic acid was administered, without the patient experiencing any symptoms. As the prescribing error was quickly identified, no additional doses were administered, and the antibiotic therapy was replaced by levofloxacin. The patient was informed of the prescribing error.

**Table 1:** Biomarkers during and after anaphylaxis.

	Standards	*T* = h0^a^	*T* = h1	*T* = h2	*T* = d5
Tryptase (µg/L)	<11	81	132	79.9	5.5
Histamine (nmol/L)	<10	>98	>98	ND	ND

h, hour; d, day; IgE, immunoglobulin E; ND, not done.

^a^ Blood sample taken 20 min after the onset of shock.

The allergology consultation carried out 4 weeks later revealed that the patient had been taking amoxicillin (3 g/day for 4 days), which had been well tolerated 1 month before the event, due to a urinary tract infection. Skin prick tests (SPTs) and intradermal skin tests (IDTs) for amoxicillin, amoxicillin/clavulanic acid, cefazolin, piperacillin-tazobactam, and cefotaxime; an SPT for latex; and basophil activation tests (BATs) (expression of CD63/CD203c by flow cytometry) for amoxicillin, amoxicillin/clavulanic acid, cefazolin, piperacillin-tazobactam, and cefotaxime were performed. The results confirmed an IgE-mediated allergy to amoxicillin and possibly clavulanic acid (Table 2): IDT and BAT results were positive for amoxicillin and amoxicillin/clavulanic acid and negative for all other drugs. A challenge with 3,000 mg of piperacillin-tazobactam was negative. Tests with clavulanic acid alone have not been carried out so far, and therefore, we cannot exclude an allergy to this molecule. Tests were performed with intravenous antibiotics used in routine practice and dispensed by our hospital pharmacy or with a commercial extract for latex (stock solutions listed in Table 2).

**Table 2:** Allergological workup carried out 4 weeks after the incident.

	SPT	IDT 10-3	IDT 10-2	IDT 10-1	BAT^b^	Specific IgE
Amoxicillin (stock solution: 20 mg/mL)	—	—	**+** **6 mm/20 mm** ^a^	ND	15%	Negative (<0.1 kU/L)
Amoxicillin-clavulanic acid (stock solution: 20 mg/mL)	—	—	**+** **6 mm/20 mm** ^a^	ND	15%	NA
Cefazolin (stock solution: 2 mg/mL)	—	ND	—	—	3%	NA
Piperacillin-tazobactam (stock solution: 20 mg/mL)	—	ND	—	—	3%	NA
Cefotaxime (stock solution: 2 mg/mL)	—	—	—	—	4%	NA
Latex (ALK-Abelló extract)	—	NA	NA	NA	NA	Negative (<0.1 kU/L)

SPT positive control: 4 mm/15 mm. SPT negative control: 0 mm/0 mm. The results are positive because of the size of the wheal and the flare compared with the controls.

mm, millimeter, NA, not applicable; ND, not done.

^a^ Size of the wheal in mm/size of the flare in mm.

^b^ BAT positive control: 93%; BAT negative control: 3%. On the basis of the concentrations tested, the result is positive for amoxicillin and amoxicillin/clavulanic acid and negative for the other molecules tested.

Amoxicillin and clavulanic acid were considered responsible for the perioperative anaphylactic shock, and a diagnosis of postanaphylactic mast cell anergy was proposed to explain the absence of clinical reaction to the reintroduction of 1 g of amoxicillin/clavulanic acid on day 3 after anaphylaxis.

## 3 Discussion

We report a case of a patient with perioperative grade 4 anaphylaxis to amoxicillin/clavulanic acid with re-exposure to the molecule 72 h later without any reaction. The patient had become sensitized to amoxicillin during a treatment received 1 month before the event. The results of positive skin tests and BATs carried out 1 month after the anaphylaxis confirmed the sensitization. The absence of an allergic reaction when 1 g of amoxicillin/clavulanic acid was reintroduced orally 3 days after anaphylaxis can be explained by a transient mast cell and potentially basophil anergy and natural desensitization. A similar case of postanaphylactic re-exposure to amoxicillin/clavulanic acid has been reported in the literature in a patient who took a 7-day course of oral amoxicillin/clavulanic acid without any reactions, 4 weeks after anaphylactic shock induced by 1.2 g of intravenous amoxicillin/clavulanic acid (9).

The antigenic epitopes of beta-lactams are thought to be related to the R1 chain, and while cefazolin, piperacillin, and cefotaxime can be used if there is a negative skin test result, in our patient, cefadroxil, cefprozil, and cefatrizine (with a shared R1 chain) (10), along with clavulanic acid, should be avoided. The use of penicillin and ampicillin will require further testing. Tryptase was found 25-fold higher than baseline and histamine was over 10 times higher within 60 min of the event, providing an insight into the mechanism of the reaction and the extent of mast cell and basophil activation.

Several studies have evaluated mast cell anergy using skin tests (11).

In 1997, Goldberg and Cofino-Cohen (1) performed SPTs, IDTs, and specific IgE tests on patients with a history of anaphylaxis to hymenoptera venom, at 1 week and then 4–6 weeks after the incident. Of the 38 patients tested, 9 had negative skin test results at the first visit but positive specific IgE test results. However, all the skin test results of these 9 patients became positive at the second visit. In 2013, Lafuente et al. (2) conducted a similar study (without specific IgE tests) on 25 patients with perioperative anaphylaxis in whom the offending allergen was identified. Ten of these patients had initial skin tests (performed between 0 and 4 days after the incident) whose results were negative and then positive at the second visit (4–8 weeks later). Several factors such as the nature of the allergen, the severity of the index reaction, and the genetic factors of patients are likely to influence mast cell mediator depletion and skin test reactivity.

**Figure 1:**
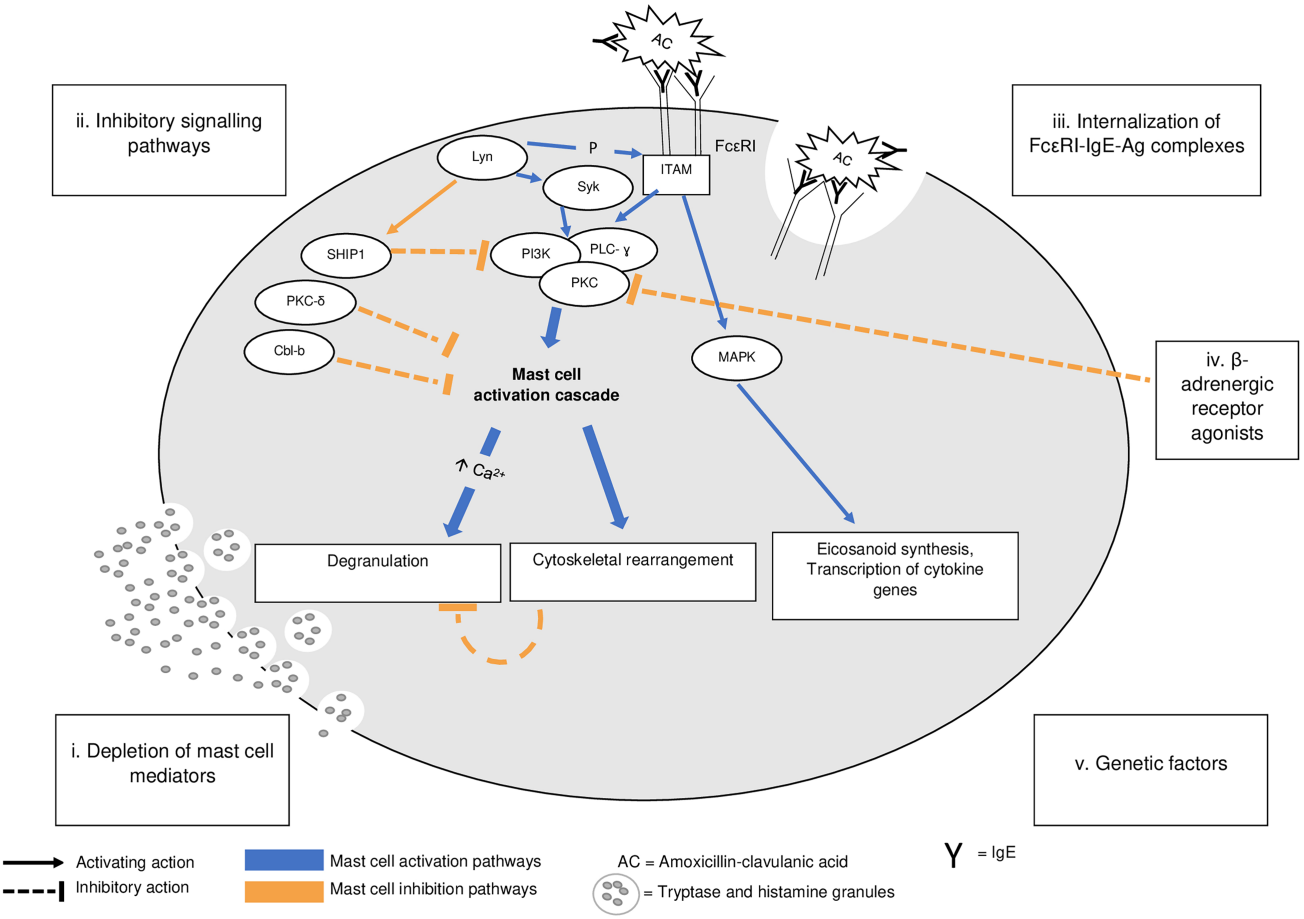
A pathophysiology of IgE-mediated mast cell activation and of postanaphylaxis mast cell anergy. Adapted from Mohamed et al. (11). IgE-dependent mast cell activation (blue labeling). Upon antigen cross-linking of the IgE/FcεRI complex, *β* and ɣ chains of the receptor aggregate and their immunoreceptor tyrosine–based activation motifs (ITAM) are phosphorylated by Lyn, which also activates Syk. This leads to a cascade involving numerous intermediates such as PI3K, PLC- ɣ, and PKC. The influx of extracellular calcium (Ca^2+^) leads to mast cell degranulation, internalization of the Ag/IgE/FceRI complexes, and cytoskeletal movements. ITAM phosphorylation is also involved in the activation of MAPK, resulting in eicosanoid synthesis and cytokine gene transcription. Postanaphylactic mast cell anergy (orange labeling). Massive degranulation can lead to a depletion of mast cell mediators, requiring a period for synthesis and regranulation. It is possible that Lyn can activate inhibitory molecules such as SHIP-1, a phosphatase inhibiting the mast cell response via the PI3K pathway. The internalization of antigen-IgE-FcεRI complexes can further reduce the number of FcεRIs available at the cell surface, impairing mast cell activation, while β-adrenergic receptor agonists can block mast cell degranulation via an inhibitory action on PKC. There is no evidence that anergy is not a universal phenomenon. Ag, antigen; FcεRI, high-affinity receptor for IgE; ITAM, immunoreceptor tyrosine–based activation motif; Lyn, member of the Src family of protein tyrosine kinases; MAPK, mitogen-activated protein kinase; P, phosphorylation; PI3K, phosphoinositide 3-kinase; PKC, protein kinase C; PLC-ɣ, phospholipase C-ɣ; SHIP 1, Src homology 2-containing inositol polyphosphate-5’-phosphatase 1; Syk, spleen-associated tyrosine kinase.

Several mechanisms are thought to be involved in postanaphylactic mast cell anergy (Figure 1): (i) mast cell regranulation after massive release of mast cell mediators; (ii) the activation of a signalosome inhibiting mast cell activation; (iii) changes in FcεRI (high-affinity IgE receptor) availability and signaling following internalization of the allergen/IgE/FcεRI complexes after anaphylaxis; (iv) external factors such as the introduction of β-adrenergic therapies blocking mast cell activation, as in the case of our patient.

After activation, mast cells do not undergo apoptosis and need time to perform a biosynthesis of mediators to regranulation and to become functional again. Mast cell “recovery” times can vary from 24 to 48 h for IL-6 and IL-13 gene expression and release of sB-hexosaminidase (12, 13) to a few days for endocytosis and granule recycling capacity (14). Hammel et al. (15) observed a significant decrease in the size of rat mast cells after strong IgE-dependent stimulation, followed by a gradual recovery of the mast cell size and their granules over 4–5 weeks. Cytoskeletal changes also appear to be involved in mast cell anergy. Seagrave and Oliver showed that overstimulated mast cells retained degranulation capacity in an environment containing inhibitors of actin polymerization (16), supporting the hypothesis that cytoskeletal rearrangements and poststimulation actin polymerization would play an inhibitory role in hypothetical second degranulation.

The internalization of allergen/IgE/FcεRI complexes following anaphylaxis leads to FcεRI membrane depletion, which can last several days, explaining mast cell anergy despite the presence of specific IgEs and allergens (17).

Repeated IgE mast cell activation with low-dose allergens induces profound quantitative and qualitative changes in Fc*ε*RI-dependent signaling (known as Fc*ε*RI desensitization), resulting in a state of unresponsiveness whose mechanisms have been analyzed in the induction of rapid drug tolerance (acute drug desensitization) and involve the activation of inhibitory phosphatases such as SHIP-1 (src homology 2-containing inositol phosphatase) (18). The suppression of mast cell responses secondary to exposure to increasing concentrations of allergens is due to the activation of inhibitory signaling pathways (19). An inhibitory signalosome with SHIP-1 (20), Lyn (src kinase family) (21), protein kinase C- δ (PKC- δ) (22), and Cbl family proteins such as Cbl-c (23) (Figure 1) has been proposed. As allergen concentrations increase, the proteins making up this signalosome are recruited. Mice or cells deficient in these various enzymes or inhibitory proteins do not develop inhibition of mast cell activation at supraoptimal allergen doses (20–24). A description of the change from a positive to a negative skin test result in a patient allergic to carboplatin after desensitization provides evidence that mast cell inhibitory mechanisms do not need massive release of mediators (25).

Studies have shown that β-adrenergic receptor agonists inhibit IgE-dependent histamine release by human mast cells (26) and that exposing healthy volunteers to salmeterol and terbutaline attenuates the cutaneous mast cell response for up to 24 h postexposure (27). However, it is not known whether these inhibitory effects follow a dose–response curve nor whether they are durable with molecules such as adrenaline, the first-line treatment for anaphylaxis and used in large quantities, as in our patient. Systemic corticoids, on the other hand, do not appear to be involved in inhibiting the mast cell response and the release of mediators (28, 29).

Postanaphylactic mast cell anergy affects mast cells in all tissues, which explains the negativity of skin test results and the absence of clinical hypersensitivity reactions on re-exposure to the allergen in the days and weeks following systemic mast cell activation. The duration of this anergy has been defined as 4–8 weeks, based on studies of skin test result positivity after anaphylaxis. However, practitioners should be aware that this duration may be longer in some patients, as suggested by the recently published clinical case in which skin test results remained negative 8 weeks after the initial accident (9). The workup carried out 4–8 weeks after anaphylactic shock should be repeated if the result is negative. Clinicians may be able to explore this window of opportunity by allowing drug-allergic patients to tolerate the culprit drug if given within a short time after anaphylaxis while the skin test result is still negative.

A 1996 study raised the possible influence of the drug's route, based on the clinical case of a patient in whom an anaphylactic reaction had been induced during a challenge with oral cloxacillin but not with intravenous cloxacillin (30). Chromatography showed no significant difference in the qualitative composition of these two drugs. Skin tests and specific IgEs performed on the patient showed negative results. The authors’ main hypothesis in their study was the formation of metabolites or different determinants from the initial molecule due to the pH conditions of the gastric barrier. Although the case of our patient is different (skin test results were positive), such a possibility should be taken into consideration as a potential alternative to explain amoxicillin tolerance in our patient.

## 4 Conclusion

In this study, postanaphylactic mast cell anergy is described in a patient allergic to amoxicillin/clavulanic acid, leading to natural desensitization at the time of accidental reintroduction of the drug within 3 days of the initial event. Research is needed to further understand this window of opportunity in patients in need of first-line therapy.

## Data Availability

The original contributions presented in the study are included in the article/Supplementary Material, further inquiries can be directed to the corresponding author.
